# Health Tract, No. 143

**Published:** 1865

**Authors:** 


### HEALTH TRACT, No. 143.

### DYING EASILY.

THE most complicated machinery, if properly made and handled, will work smoothly, easily, and well, until it is worn entirely out, all its parts having been worked equally; but if it had met with constant shocks and jars and strains, or if a stone had been thrown among its wheels, an early or violent disruption of all the parts would have been an inevitable result. Thus is it with the greatest of all mechanisms, the human body, whose builder was Omnipotence; it, too, follows the great law, the more equally and regularly it is worked; the more care is taken of it, the longer will it last; and its ending will come so gently, that it can scarcely be told by the stop-watch at what point of time the workings of life have ceased forever. It is uniformly thus with those who die at an advanced age; and these are they who, either by instinct or reason, or a fortunate induction in early life into habits of regularity and moderation, and quietude and serenity, have prevented the shocks and jolts and jars which, in other cases, have broken up the bodily machinery before it has half worn out. It is the unequal working of single wheels of life which brings premature decay and dreadful, agonizing deaths. To the good man, it is distinctly promised: "Thou shalt come to thy grave in a full age, like as a shock of corn cometh in, in his season." Not so the glutton, who taxes his stomach to its highest capacity; not so with the effeminate, who deprives the body of its wonted exercise, and thus puts a clog on its wheels, while the involuntary organs, those over which he has no direct control, work on in their natural rapidity; not so with the licentious, who run riot in their abandonment to animal appetite; these all die before their time, and their last sickness is painful and long, extending through weeks and months and weary years. But the very old are passing away from us all the time, of whom it is said, they were apparently as well as ever the day, and even the hour, before they died, and life went out as gently as the failing embers on the hearth. The genial and kindly-remembered "Laurie Todd," (Grant Thorburn,) a model of temperance, regularity, cheeriness, and industry, was standing by the fire in pleasant conversation with his worthy wife one minute, and in almost the next, he had passed on to his great account. His widow wrote to us a few days after his peaceful departure: "I do thank you especially for the March number; the testimony of the wise and good to the piety and virtue of dear Grant being just like a cordial to me in my hour of sorrow. He went out to see the thermometer, with his glasses on, forgetting I had sent it away the day before, for a little repair. The last words he said were, 'No danger,' alluding to my caution that he would burn his coat, as he was standing by the fire; the last words he ever wrote, 'lay down and went to sleep,' only thirty-six hours before his decease. So it may be said of him, that he wrote to the last; although he could not see to read for the last two or three years of his life. I never heard him murmur or complain. He viewed the hand of God in every event of life. He was a great admirer of Washington, and his last conversation was of his funeral-procession in New-York, on the last day of 1799. His mind was clear, his voice so strong, that I little thought what the morrow would bring forth. God bless you, Dr. Hall, and may you and I, and all our dear friends, meet around the Throne. Thine sincerely, M. C. THORBURN." N. B. He died aged ninety-one years.

Thus, peacefully, quietly, without pain, do the aged and the good, who have lived temperately, industriously, and genially, almost always pass away. Let us, reader, live as they, that our end may be like theirs!

